# GutUDB: A comprehensive multiomics database for intestinal diseases

**DOI:** 10.1002/imt2.195

**Published:** 2024-04-27

**Authors:** Yi Bao, Yaxin Chen, Lizhu Lin, Jingyi Li, Xinli Liu, Gang Wang, Yueqi Li, Yao Lin, Yajing Chen, Lijuan Zhou, Yawen Qi, Yufang Xie, Zhenrui Lin, Zhe Sun, Yuwen Fan, Jinjing Jiang, Feiyu Zhang, Hubin Chen, Jiemei Chu, Jiegang Huang, Xuena Chen, Hao Liang, Shuaiyi Liang, Sanqi An

**Affiliations:** ^1^ Guangxi Key Laboratory of AIDS Prevention and Treatment & Guangxi Colleges and Universities Key Laboratory of Prevention and Control of Highly Prevalent Diseases, School of Public Health Guangxi Medical University Nanning China; ^2^ Institute of Respiratory Health, Frontiers Science Center for Disease‐related Molecular Network, West China Hospital Sichuan University Chengdu China; ^3^ Department of Anesthesiology The First People's Hospital of Qinzhou Qinzhou China; ^4^ Department of Pathology Guangdong Second Provincial General Hospital Guangzhou China; ^5^ Precision Medicine Translational Research Center, West China Hospital Sichuan University Chengdu China; ^6^ Department of Biochemistry and Molecular Biology, School of Basic Medicine Guangxi Medical University Nanning China; ^7^ Life Sciences Institute Guangxi Medical University Nanning China; ^8^ The Key Laboratory of Experimental Teratology, Ministry of Education, Department of Systems Biomedicine, School of Basic Medical Sciences Shandong University Jinan China; ^9^ Department of Bioinformatics Anjin Biotechnology Co., Ltd. Guangzhou China

## Abstract

Gut Universe Database (GutUDB) provides a comprehensive, systematic, and practical platform for researchers, and is dedicated to the management, analysis, and visualization of knowledge related to intestinal diseases. Based on this database, eight major categories of omics data analyses are carried out to explore the genotype‐phenotype characteristics of a certain intestinal disease. The first tool for comprehensive omics data research on intestinal diseases will help each researcher better understand intestinal diseases.

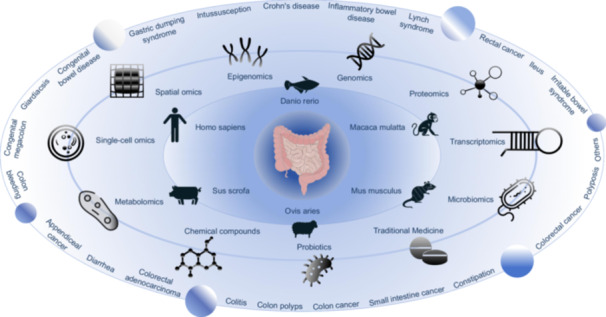

The intestine serves as the central organ in the digestive system for nutrient absorption and digestion processes [1]. In recent years, high‐throughput sequencing technologies have significantly broadened our comprehension of biological mechanisms from diverse aspects, including DNA‐level processes, transcriptional dynamics, protein‐related activities, and epigenetic modifications. These various omics sequencing data could provide a more systematic and better insight into intestinal diseases [2, 3]. However, the complexity of analytical techniques for sequencing data, coupled with the dispersed nature of data storage and the voluminous size of data sets, has impeded researchers to fully exploit these omics resources [4, 5]. Therefore, the establishment of a comprehensive database to integrate and analyze these sequencing data sets would be urgent and instrumental in dealing with these challenges.

Here, we present the Gut Universe Database (GutUDB), a high‐quality and comprehensive multiomics database about intestinal diseases. It provides a user‐friendly platform for the comprehensive collection of eight types of omics data, including epigenomics, genomics, transcriptomics, spatial omics, single‐cell omics, proteomics, metabolomics, and microbiomics data, spanning 56 distinct intestinal diseases across six various species. GutUDB offers a thorough analysis of intestinal diseases, presenting omics data through various informative charts. We also highlight the therapeutic targets for both chemical and traditional medicine suitable to intestinal diseases, along with the associated therapeutic outcomes involving probiotics. GutUDB will play a pivotal role in identifying diagnostic targets for intestinal diseases and unveiling the molecular mechanisms underlying the progression of these conditions.

## RESULTS

### Overview of GutUDB

**Figure 1:**
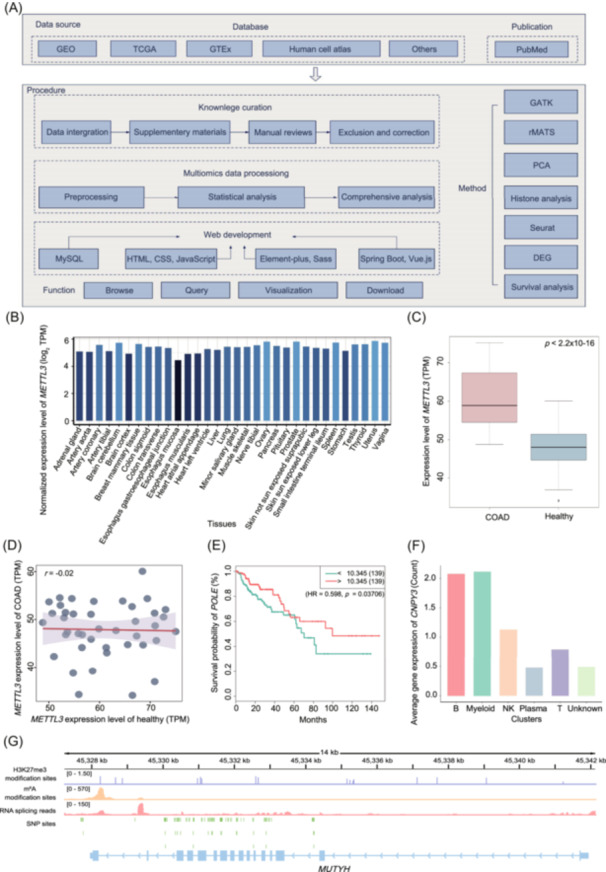
Gut Universe Database (GutUDB) contents and features. (A) The process of data collection and processing, as well as the construction procedure of the database website, incorporating four core functions (browsing, querying, visualizing, and downloading). (B) The expression levels of the *METTL3* in different tissues. The data derived from GTEx. (C) Boxplot displaying gene expression difference between COAD and healthy tissues. *p* < 0.05 indicated that the expression of *METTL3* was significantly different between the two groups. (D) Scatter plot showing the correlation analysis of *METTL3* expression between COAD group and healthy group. *r* = −0.02 indicated that *METTL3* expression almost no correlation between the two groups. (E) Survival analysis curve of *POLE* associated with COAD. (F) The expression levels of *CNPY3* in various cells and the presentation of expression maps in scRNA‐seq data. (G) Tracks displaying the read coverage of H3k27me3, the m^6^A sites as well as RNA‐seq and SNP data in colorectal cancer on the *MUTYH*. B, B lymphocyte; COAD, colon adenocarcinoma; CSS, cascading style sheets; DEG, differential expression analysis; GEO, Gene Expression Omnibus; GTAK, Genome Analysis Toolkit; GTEx, Genotype‐Tissue Expression project; HR, hazard ratio; HTML, HyperText Markup Language; m^6^A, N6‐methyladenosine; MySQL, My Structured Query Language; NK, natural killer cell; Plasma, Plasma cell; PCA, principal component analysis; r, correlation coefficient; Sass, Syntactically Awesome Stylesheets; SNP, single‐nucleotide polymorphism; T, T lymphocyte; TCGA, The Cancer Genome Atlas; TPM, Transcripts Per Million.

To date, GutUDB has accumulated approximately 9 million generated profiles from eight types of omics data sets, which encompasses 56 intestinal diseases across six species: *Homo sapiens, Mus musculus, Rattus norvegicus, Macaca mulatta, Danio rerio, and Sus scrofa*. Overall, GutUDB incorporated 58,970 genes derived from 11 subtypes of intestinal tissue or 63 intestinal cell lines, and identified various potential clinical therapeutics, including chemical drugs, traditional medicine, and probiotic agents. To assist users to easily elucidate and interrogate the intricate gene‐disease‐omics network, four core functionalities—Browse, Query, Visualization, and Download—were integrated into GutUDB (Figure 1A).

### Disease‐gene associations across distinct intestinal diseases

In total, 260,790 disease‐gene associations were embedded in GutUDB. The main intestine‐related diseases, including colon cancer, colorectal cancer (CRC), bleeding of the colon, rectal cancer, constipation, diarrhea, ileus, inflammatory bowel disease, and small intestine cancer (Supporting Information: Figure S1A), shared common genes such as *STK11*, *CFTR*, *BMPR1A*, *SMAD4*, *NOTCH1*, *PKD1*, *MLH1*, *MSH2*, *APC*, and *MEFV* (Supporting Information: Figure S1B) [6, 7, 8, 9, 10, 11]. Specifically, *STK11* is a well‐known etiological factor for Peutz‐Jeghers syndrome. *SMAD4* and *BMPR1A* are reported to be associated with Juvenile Polyposis Syndrome [12]. These findings illustrated the strong relationship between intestinal pathologies and these genes.

To further explore specific genes related to intestinal diseases, users can easily obtain detailed information on the homepage using gene symbols. For example, *METTL3*, upon clicking the “Search” icon, the results exhibited the details of this gene, such as genome location and functional characteristics (e.g., RNA‐binding protein or transcription factor) (Supporting Information: Figure S1C), the expression levels and patterns in different tissues across eight omics levels (Figure 1B). We observed a frequency of copy number variation (CNV) deletion of only 0.04 for *METTL3* in CRC, while there was a significantly high RNA expression of this gene. We concluded that there is a weak correlation between CNV RNA expression on *METTL3*, *similar* correlation analyses can also be conducted for other epigenetic and proteomics data (Supporting Information: Figure S1D). Additionally, GutUDB specifically offered information on gene sets with differential expression and survival prognosis in colon adenocarcinoma (COAD) patients (Figure 1C–E).

### Four distant models: Disease, treatment, species, and omics data

GutUDB was primarily divided into four major modules—SPECIES, DISEASES, OMICS, and THERAPY, facilitating users to access and browse corresponding details upon clicking each icon on the homepage. In the “THERAPY” module, GutUDB curated 21,984 drug‐disease interactions, including 6281 chemical compounds, 393 traditional medicinal herbs, and 22 probiotics (Supporting Information: Figure S1E). Among these associations, cisplatin, as a chemotherapy drug for colon cancer patients, could infiltrate tumor cells, induce DNA damage, and ultimately lead to cell death [13]. It was demonstrated cisplatin had strong connectivity in the drug‐disease‐gene network, highlighting the reliability and accessibility of the information stored in GutUDB. In the “DISEASES” module, users can take deeper insight into genes of interest associated with various intestinal disorders across different omics levels (Supporting Information: Figure S1F). In the “SPECIES” module, users can browse all genes and select species they are interested in (Supporting Information: Figure S1G). All results were presented in a tabular format and facilitated users to efficiently retrieve and filter them by inputting keywords (e.g., a gene symbol or a specific type of intestinal disease) or by clicking on terms (e.g., omics level or hot genes) at the top of the current page.

### Browsing spatial omics and single‐cell omics data of intestinal diseases

Recently, spatial omics and single‐cell sequencing methods have significantly advanced the study of cellular heterogeneity, immune regulation, and molecular mechanisms in intestinal diseases. The spatial omics data in GutUDB revealed intricate spatial expression maps delineating tumor‐specific genes across diverse samples, along with annotation maps. Upon selecting a gene, users could peruse gene‐related information of spatial omics data in GutUDB. Furthermore, we also provided details about sample, tissue type, biotechnology, and the gene's expression profiles of spatial omics data in GutUDB. The homepage of gene expression showed the uniform manifold approximation and projection plots of single‐cell RNA‐seq, and each sample's detail page contained the expression maps of the genes in different cells (Figure 1F). Besides, single‐cell gene expression, single‐cell alternative polyadenylation, single‐cell alternative splicing, and single‐cell proteomics data related to intestinal diseases were also embedded in GutUDB, facilitating users to comprehensively understand regulatory mechanisms from different dimensions such as RNA and proteins at the single‐cell level.

### Interactive visualization of bulk multiomics profiles related to intestinal diseases

To facilitate the integration and analysis of diverse data sets and data types, we have combined diverse omics data types with the complex interplay among DNA, RNA, proteins, and other aspects of genetic turbulence. Users can assess a specific omics type through “Omics” option in the navigation bar. Eight different types of bulk omics were integrated into GutUDB for interactive visualization.

Epigenetics plays a crucial role in the development and progression of intestinal diseases, and researchers have screened and utilized epigenetic molecules as diagnosis and prognosis biomarkers in clinical trials [14]. In GutUDB, three mainly epigenomics were embedded, including DNA methylation, histone modification (H3K27me3, H3K27ac, H3K36me3, H3K4me1, H3K4me3, and H3K9me3), and chromosome structure. Among them, the transcriptional and posttranscriptional regulatory mechanisms involving RNA m^6^A modification and alternative splicing, are the focal points of current research. Here we demonstrate the histone modification status, specifically the H3K27me3 modification, using *MUTYH* as an example (Figure 1G).

Based on miCLIP‐seq, 1908 RNA m^6^A modification sites were included in GutUDB. As for genomics, alterations can influence RNA modification and result in gene transcript level alteration, which may consequently affect the protein expression levels. Additionally, six types of alternative splicing were also incorporated into the GutUDB database, including exon skipping, alternative 5′splice site, alternative 3′splice site, mutually exclusive exons, and retained introns [15]. Here, we use *MUTYH* as an example to demonstrate the status of alternative splicing and m^6^A modification in CRC (Figure 1G).

For genomics, GutUDB currently includes 72,248 single‐nucleotide polymorphisms (SNPs), 54,131 CNVs, 1097 structural variations of chromosome (SVs), and 92,888 mutated genes related to different intestinal diseases, details were established on the “Statistics” page. Notably, each genomic alternation was accompanied by its frequency in various populations as well as data set source. Here, we present the SNP sites on the *MUTYH* gene in CRC (Figure 1G).

For transcriptomics, 137 RNA‐seq data sets related to 7.9 million transcriptional profiles with distinct gene expression patterns under various conditions or in different tissues were collected (Supporting Information: Figure S1H). Besides, noncoding RNAs associated with intestinal diseases were separately displayed as panels, including 62 circular RNAs, 182 long noncoding RNAs, and 58 microRNAs.

For proteomic and metabolomic profiles, aberrations significantly have impact on the pathophysiology of intestinal diseases [16, 17]. In the GutUDB, the proteomics data provided information of protein characteristics including functional domains, active sites, and posttranslational modifications under artificial regulation. Moreover, data from metabolomics and microbiomics demonstrated 2764 relationships between gut microbes and metabolites in GutUDB.

Therefore, GutUDB enabled researchers to conduct a deep study into the complex interconnections of genetic modifications from multiple biological levels and insights, thus unveiling the sophisticated processes underlying gene regulation.

## DISCUSSION AND FUTURE DIRECTION

In this study, the main challenge was the standardization of integrating sequencing data across different platforms, such as RNA‐seq and microarray data. Admittedly, there are variations in sequencing platforms and instruments. Our database GutUDB mainly utilizes RNA‐seq data for bulk transcriptomics due to it can still be standardized and compared effectively. Integrating diverse omics data, including transcriptomics, can illuminate pathogenic changes and significantly enhance our understanding of disease diagnosis, mechanisms, and treatment strategies [18]. GutUDB will definitely serve as a comprehensive resource for a wide range of users, such as clinicians specializing in gastroenterology, researchers in academic and scientific institutions, educators and students from universities and anyone with an interest in intestinal research and clinical applications. Users can easily get access to an extensive array of omics data related to intestinal diseases directly through GutUDB, without exhaustive searches for disease‐specific information across various databases, such as noncoding RNA databases, spatial omics repositories, and microbiome databases.

With the rapid accumulation of diverse sequencing data, we are responsible for frequently updating GutUDB, and proudly announce that the GutUDB is dedicated to serving as an open‐access resource for the global community to advance the study of intestinal diseases. We also plan to integrate interactive models into GutUDB within the next 1–2 years, facilitating real‐time communication between users on the platform. We will also integrate a more comprehensive collection of pathological slides on intestinal diseases, radiomics of intestinal diseases, and cohorts of individuals with intestinal diseases into GutUDB. All in all, these procedures will ensure GutUDB obtains the newest and latest extensive multiomics database for intestinal diseases.

## AUTHOR CONTRIBUTIONS

Sanqi An, Shuaiyi Liang, HaoLiang, and Xuena Chen conceived and designed the study, revised the manuscript, and prepared it for publication. Yi Bao, Yaxin Chen, Lizhu Lin, and Jingyi Li carried out the implementation and supervision of the project, collected data, analyzed the results, built the website, interpreted the results, and wrote the manuscript. Gang Wang and Xinli Liu performed the data collection and analysis. Yueqi Li and Yao Lin assisted in database development and application. Yajing Chen and Lijuan Zhou organized and reviewed the omics data. Yawen Qi and Yufang Xie excluded and corrected the collected data. Zhenrui Lin, Zhe Sun, Yuwen Fan, Jinjing Jiang, and Feiyu Zhang collected and organized part of the omics data. Hubin Chen, Jiemei Chu, and Jiegang Huang conducted the search and integration of relevant literatures. All authors have read the final manuscript and approved it for publication.

## CONFLICT OF INTEREST STATEMENT

The authors declare no conflict of interest.

## ETHICS STATEMENT

No animals or humans were involved in this study.

## Supporting information


**Figure S1**: Web display of content from other sections.

**Table S1**: Statistical source data.

## Data Availability

All data sources analyzed in this study are included in the Supporting Information (Table S1). GutUDB is freely available at https://intestine.splicedb.net. The code involved in this research and the data tables corresponding to the figures in the article have been uploaded to GitHub https://github.com/Ansanqi/GutUDB. Supporting Information (methods, figures, tables, scripts, graphical abstract, slides, videos, Chinese translated version, and updated materials) may be found in the online DOI or iMeta Science http://www.imeta.science/.
