# Supplementary material for: GutUDB: A comprehensive multiomics database for intestinal diseases
Source: Imeta. 2024 Apr 27;3(3):e195. doi: 10.1002/imt2.195 (PMC11183169; doi:10.1002/imt2.195)
Supplement: Supplementary file 1 — Figure S1: Web display of content from other sections. [file IMT2-3-e195-s002.docx]

# Supporting information to

**GutUDB：A comprehensive multi-omics database for intestinal diseases**

**Running Title: Introduction to the content and use of the GutUDB**

Yi Bao^1#^, Yaxin Chen^2#^, Lizhu Lin^3#^, Jingyi Li^4#^, Xinli Liu^1^, Gang Wang^5^, Yueqi Li^1^, Yao lin^1^, Yajing Chen^6^, Lijuan Zhou^1^, Yawen Qi^2^, Yufang Xie^2^, Zhenrui Lin^7^, Zhe Sun^7^, Yuwen Fan^7^, Jinjing Jiang^7^, Feiyu Zhang^7^, Hubin Chen^1^, Jiemei Chu^1^, Jiegang Huang^1^, Xuena Chen^8*^, HaoLiang^1*^, Shuaiyi Liang^9*^, Sanqi An^1*^

^1^Guangxi Key Laboratory of AIDS Prevention and Treatment & Guangxi Colleges and Universities Key Laboratory of Prevention and Control of Highly Prevalent Diseases, School of Public Health, Guangxi Medical University, Nanning 530021, China

^2^Institute of Respiratory Health, Frontiers Science Center for Disease-related Molecular Network, West China Hospital, Sichuan University, Chengdu 610041, China

^3^The First People’s Hospital of Qinzhou, Qinzhou 535000, China

^4^Department of pathology, Guangdong Second Provincial General Hospital, Guangzhou 510317, China

^5^Innovation Laboratory for Precision Diagnostics, Precision Medicine Research Center, Precision Medicine Key Laboratory of Sichuan Province, West China Hospital, Sichuan University, Chengdu 610041, China

^6^Department of Biochemistry and Molecular Biology, School of Basic Medicine. Guangxi Medical University, Nanning 530021, China

^7^Life Sciences Institute, Guangxi Medical University, Nanning 530021, China

^8^The Key Laboratory of Experimental Teratology, Ministry of Education, Department of Systems Biomedicine, School of Basic Medical Sciences, Shandong University, Jinan 250012 China

^9^Department of bioinformatics, Anjin Biotechnology Co., Ltd., Guangzhou 510000, China

^#^These authors contributed equally: Yi Bao, Yaxin Chen, Lizhu Lin, and Jingyi Li

^*^Correspondence: [ansq@mail2.sysu.edu.cn](mailto:ansq@mail2.sysu.edu.cn) (Sanqi An), [lianghao@gxmu.edu.cn](mailto:lianghao@gxmu.edu.cn) (Hao Liang),

[SLiang2@my.harrisburgu.edu](mailto:SLiang2@my.harrisburgu.edu) (Shuaiyi Liang), [chenxuena2021@sdu.edu.cn](mailto:chenxuena2021@sdu.edu.cn) (Xuena Chen).

**METHOD**

**Multiomics Data collection and collation**

We identified genes associated with the intestinal diseases through the following methods, (1) By analyzing multi-omics data from case and control groups, we determined which genes are associated with the disease at different omics levels. For different omics data, epigenomic information related to intestinal diseases was obtained from Genome Sequence Database (GSDB) [1], MethBank [2], and the ENCODE Portal [3]. Genomic data was collected from the cBio Cancer Genomics Portal [4], NCBI ClinVar (<https://www.ncbi.nlm.nih.gov/clinvar/>), and Online Mendelian Inheritance in Man (OMIM) [5]. Proteomic insights were derived from Uniprot [6]. (2) Text mining was also employed to identify genes previously proven to be related to intestinal diseases. Keywords depicting diverse intestinal sites such as "colon", "sigmoid colon", "duodenum", and intestinal diseases such as "colon cancer", "colitis", and "rectal cancer", were systematically collected from these databases. To be more specific, we conducted a thorough review of 2150 articles about intestinal disease from PubMed (<https://pubmed.ncbi.nlm.nih.gov/>) and extracted 58,970 disease-related genes, 260,790 disease-gene associations and 21,983 gene-targeted therapies from several muti-omics databases initially. (3) A targeted retrieval from different single-cell omics databases was conducted to find genes related to intestinal diseases, with the search primarily based on keywords. Single-cell omics data retrieved from the IMMUcan SingleCell RNAseq Database (<https://immucanscdb.vital-it.ch/>) and scAPAdb [7]. Spatial omics data was curated from the Spatial Omics DataBase (SODB) [8]. Dysregulation of RNA processes such as RNA splicing and editing, non-coding RNA, and RNA methylation data were gathered from COVID-19 Alternative Splicing Atlas (CASA) [9], miR2Disease [10], LncRNADisease [11], and RMVar [12]. To further discover metagenomic about intestinal diseases, we systematically queried the NCBI BioProject (<https://www.ncbi.nlm.nih.gov/bioproject/>) to make sure accuracy in classifying them as transcriptome datasets. Additionally, metagenomic and 16S data collected from the projects within gutMGene and mbodymap were under the screen of microorganisms present in the large and small intestines [13,14]. The Encyclopedia of Traditional Chinese Medicine (ETCM) with traditional medicinal knowledge were utilized to address conditions associated with intestinal diseases [15]. Besides, Chemical-gene-disease interactions were extracted from the Comparative Toxicogenomics Database (CTD) [16]. Data on probiotics was collected from ProbioticsDB (<https://probioticsdb.com/>).

**Analysis DEGs and tissue special genes of transcriptomics**We downloaded raw transcriptomics and clinical data from 165 projects in Gene Expression Omnibus (GEO) [17], The Cancer Genome Atlas (TCGA) (<https://portal.gdc.cancer.gov/>), and Clinical Proteomic Tumor Analysis Consortium (CPTAC) (<https://cptac-data-portal.georgetown.edu/>) and then took a deeply comprehensive analysis of gene expression and prognosis. HISAT2 (v 2.1.0) and StringTie (v 1.3.4) were used to map the sequencing reads to the hg38 and mm10 genomes and calculate the Transcripts Per Million (TPMs) of genes, respectively. HTSeq was employed for the determination of read counts for each gene, followed by differential gene expression (DEGs) analyses with DESeq2. We used the quantile normalization method in the R package "preprocessCore" to normalize the gene expression levels across all samples, thereby minimizing the bias caused by different batches across the different libraries. Genes exhibiting a False Discovery Rate (FDR) exceeding 0.05 were identified as Differentially Expressed Genes (DEGs). Ultimately, the "ggplot2" package (v 2.0.0) was used to visualize the acquired datasets. The overall Kaplan–Meier (KM) survival analysis for each gene expression group was conducted using the "survfit" and "survdiff" functions within the "survival" package. We collected transcriptomic data from various tissue types in the Genotype-Tissue Expression project (GTEx) database and subsequently calculated the expression profiles of each gene in intestinal tissues compared to other tissues to identify tissue special genes.

**Analysis of RNA splicing and Alternative polyadenylation (APA) using RNA-seq data**

The sorted BAM files underwent alternative splicing analysis via rMATS (v 4.1.2) [18]. Then, the results were visualized by rmats2sashimiplot (v 2.0.4) with parameters rMATS "py --b1. /b1.txt --b2. /b2.txt --gtf. /Mus_musculus. GRCm39.105.gtf -t single --readLength 100 --nthread 30 --od. /output --tmp. /tmp_output". Alternative splicing (AS) is an important component of gene expression regulation and contributes to proteome diversity [19]. AS events analysed by maser (v 1.12.1) R package were considered to be significant when the average coverage exceeds 5, and delta percent spliced in (ΔPSI) exceeds 0.1. The long-read data for colon cancer were obtained from the Singapore Nanopore Expression Project (<https://github.com/GoekeLab/sg-nex-data> (ENA PRJEB44348)) [20].

**Organization of Single-cell omics and Spatial omics profiles**

Single-cell expression data were obtained from the IMMU Cancer Database and Cancer Single-cell Expression Map, with a particular focus on screening "colon" and "colorectal" tissues [21-27]. A total of eight datasets were eventually downloaded for the purpose of single-cell omics analysis. Genes exhibiting low-quality were excluded through a filtration process that considered the count of identified genes, mitochondrial gene content, and total counts. Uniform manifold approximation and projection (UMAP) plots were used to visualize the data in reduced dimensions, with cell types in each cluster determined based on known marker genes. In the context of spatial omics data, raw count values underwent normalization via total counts and subsequently log-transformed. In instances where datasets comprised more than 2,000 features, emphasis was placed on selecting the top 2,000 highly variable genes using the highly variable gene function (flavor='seurat'). Principal component analysis (PCA) was performed to reduce the dimensions of the data. Employing Scanpy's neighbor's function, we identified cell clusters with similar transcriptomic profiles. Consequently, the Leiden algorithm was applied to categorize the data into distinct groups.

**Database construction and web development**

All data were stored in the My Structured Query Language (MySQL) (v 5.7.32) database (<https://www.mysql.com/>). The front-end (web pages) of the website was coded by HyperText Markup Language (HTML), Cascading Style Sheets (CSS) and JavaScript and developed by Vue.js (v 3.3.4) (<https://cn.vuejs.org/>) framework. While, the back-end (services) was coded by Java (v 1.8.0) (<https://www.java.com/>) and developed by Spring Boot (v 2.3.1) (<https://spring.io>) framework in combination with Mybatis Plus (<https://baomidou.com>) low-code platform. Then, Axios (<https://www.axios-http.cn/>) for web requests and Vue-router (v 4.2.4) (<https://router.vuejs.org/>) were used to build application pages. Element-plus (v 2.3.8) (<https://element.eleme.io/>) was used for designing the user interaction interface and Syntactically Awesome Stylesheets (Sass) (v 1.63.6) (<https://sass.bootcss.com/>) for CSS styling. Vite (v 4.4.0) (<https://vitejs.cn/>) was used to build on the code. Therefore, we associated genes with other omics and therapeutic terms to make the website more interactive and available to all users. We have organized and uploaded the all the code of the website to GitHub: <https://github.com/Ansanqi/GutUDB>. Besides, relevant README file is provided to help users better understand and use the project (<https://github.com/Ansanqi/GutUDB/blob/main/Readme.docx>).

**REFERENCES**

1. Oluwadare, Oluwatosin, Max Highsmith, Douglass Turner, Erez Lieberman Aiden, Jianlin Cheng. 2020. “GSDB: a database of 3D chromosome and genome structures reconstructed from Hi-C data.” *BMC Molecular and Cell Biology* 21: <https://doi.org/10.1186/s12860-020-00304-y>

2. Zhang, Mochen, Wenting Zong, Dong Zou, Guoliang Wang, Wei Zhao, Fei Yang, Song Wu, et al. 2023. “MethBank 4.0: an updated database of DNA methylation across a variety of species.” *Nucleic acids research* 51: D208-D216. <https://doi.org/10.1093/nar/gkac969>

3. Sloan, Cricket A., Esther T. Chan, Jean M. Davidson, Venkat S. Malladi, J. Seth Strattan, Benjamin C. Hitz, Idan Gabdank, et al. 2016. “ENCODE data at the ENCODE portal.” *Nucleic acids research* 44: D726-D732. <https://doi.org/10.1093/nar/gkv1160>

4. Cerami, Ethan, Jianjiong Gao, Ugur Dogrusoz, Benjamin E. Gross, Selcuk Onur Sumer, Bülent Arman Aksoy, Anders Jacobsen, et al. 2012. “The cBio Cancer Genomics Portal: An Open Platform for Exploring Multidimensional Cancer Genomics Data.” *Cancer Discovery* 2: 401-404. <https://doi.org/10.1158/2159-8290.Cd-12-0095>

5. Amberger, Joanna S., Carol A. Bocchini, François Schiettecatte, Alan F. Scott, Ada Hamosh. 2015. “OMIM.org: Online Mendelian Inheritance in Man (OMIM®), an online catalog of human genes and genetic disorders.” *Nucleic acids research* 43: D789-D798. <https://doi.org/10.1093/nar/gku1205>

6. Bateman, Alex, Maria-Jesus Martin, Sandra Orchard, Michele Magrane, Shadab Ahmad, Emanuele Alpi, Emily H. Bowler-Barnett, et al. 2023. “UniProt: the Universal Protein Knowledgebase in 2023.” *Nucleic acids research* 51: D523-D531. <https://doi.org/10.1093/nar/gkac1052>

7. Zhu, Sheng, Qiwei Lian, Wenbin Ye, Wei Qin, Zhe Wu, Guoli Ji, Xiaohui Wu. 2022. “scAPAdb: a comprehensive database of alternative polyadenylation at single-cell resolution.” *Nucleic acids research* 50: D365-D370. <https://doi.org/10.1093/nar/gkab795>

8. 2023. “Spatial Omics DataBase (SODB): increasing accessibility to spatial omics data.” *Nature Methods* 20: 359-360. <https://doi.org/10.1038/s41592-023-01772-8>

9. Chen, Yaxin, Gang Wang, Jingyi Li, Lei Xia, Lin Zhu, Wenxing Li, Qiang Luo, et al. 2022. “CASA: a comprehensive database resource for the COVID-19 Alternative Splicing Atlas.” *Journal of Translational Medicine* 20: <https://doi.org/10.1186/s12967-022-03699-8>

10. Jiang, Q., Y. Wang, Y. Hao, L. Juan, M. Teng, X. Zhang, M. Li, G. Wang, Y. Liu. 2009. “miR2Disease: a manually curated database for microRNA deregulation in human disease.” *Nucleic acids research* 37: D98-D104. <https://doi.org/10.1093/nar/gkn714>

11. Bao, Zhenyu, Zhen Yang, Zhou Huang, Yiran Zhou, Qinghua Cui, Dong Dong. 2019. “LncRNADisease 2.0: an updated database of long non-coding RNA-associated diseases.” *Nucleic acids research* 47: D1034-D1037. <https://doi.org/10.1093/nar/gky905>

12. Luo, Xiaotong, Huiqin Li, Jiaqi Liang, Qi Zhao, Yubin Xie, Jian Ren, Zhixiang Zuo. 2021. “RMVar: an updated database of functional variants involved in RNA modifications.” *Nucleic acids research* 49: D1405-D1412. <https://doi.org/10.1093/nar/gkaa811>

13. Cheng, Liang, Changlu Qi, Haixiu Yang, Minke Lu, Yiting Cai, Tongze Fu, Jialiang Ren, Qu Jin, Xue Zhang. 2022. “gutMGene: a comprehensive database for target genes of gut microbes and microbial metabolites.” *Nucleic acids research* 50: D795-D800. <https://doi.org/10.1093/nar/gkab786>

14. Jin, Hanbo, Guoru Hu, Chuqing Sun, Yiqian Duan, Zhenmo Zhang, Zhi Liu, Xing-Ming Zhao, Wei-Hua Chen. 2022. “mBodyMap: a curated database for microbes across human body and their associations with health and diseases.” *Nucleic acids research* 50: D808-D816. <https://doi.org/10.1093/nar/gkab973>

15. Xu, Hai-Yu, Yan-Qiong Zhang, Zhen-Ming Liu, Tong Chen, Chuan-Yu Lv, Shi-Huan Tang, Xiao-Bo Zhang, et al. 2019. “ETCM: an encyclopaedia of traditional Chinese medicine.” *Nucleic acids research* 47: D976-D982. <https://doi.org/10.1093/nar/gky987>

16. Davis, Allan Peter, Cynthia J. Grondin, Robin J. Johnson, Daniela Sciaky, Jolene Wiegers, Thomas C. Wiegers, Carolyn J. Mattingly. 2021. “Comparative Toxicogenomics Database (CTD): update 2021.” *Nucleic acids research* 49: D1138-D1143. <https://doi.org/10.1093/nar/gkaa891>

17. Barrett, T., D. B. Troup, S. E. Wilhite, P. Ledoux, C. Evangelista, I. F. Kim, M. Tomashevsky, et al. 2010. “NCBI GEO: archive for functional genomics data sets--10 years on.” *Nucleic acids research* 39: D1005-D1010. <https://doi.org/10.1093/nar/gkq1184>

18. Shen, Shihao, Juw Won Park, Zhi-xiang Lu, Lan Lin, Michael D. Henry, Ying Nian Wu, Qing Zhou, Yi Xing. 2014. “rMATS: Robust and flexible detection of differential alternative splicing from replicate RNA-Seq data.” *Proceedings of the National Academy of Sciences* 111: <https://doi.org/10.1073/pnas.1419161111>

19. Zhao, Shanrong. 2019. “Alternative splicing, RNA-seq and drug discovery.” *Drug Discovery Today* 24: 1258-1267. <https://doi.org/10.1016/j.drudis.2019.03.030>

20. Chen, Ying, Nadia M. Davidson, Yuk Kei Wan, Harshil Patel, Fei Yao, Hwee Meng Low, Christopher Hendra, et al. 2021. “A systematic benchmark of Nanopore long read RNA sequencing for transcript level analysis in human cell lines.” *bioRxiv* <https://doi.org/10.1101/2021.04.21.440736>

21. Qian, Junbin, Siel Olbrecht, Bram Boeckx, Hanne Vos, Damya Laoui, Emre Etlioglu, Els Wauters, et al. 2020. “A pan-cancer blueprint of the heterogeneous tumor microenvironment revealed by single-cell profiling.” *Cell Research* 30: 745-762. <https://doi.org/10.1038/s41422-020-0355-0>

22. Zhang, Lei, Xin Yu, Liangtao Zheng, Yuanyuan Zhang, Yansen Li, Qiao Fang, Ranran Gao, et al. 2018. “Lineage tracking reveals dynamic relationships of T cells in colorectal cancer.” *Nature* 564: 268-272. <https://doi.org/10.1038/s41586-018-0694-x>

23. Zhang, Yuanyuan, Liangtao Zheng, Lei Zhang, Xueda Hu, Xianwen Ren, Zemin Zhang. 2019. “Deep single-cell RNA sequencing data of individual T cells from treatment-naïve colorectal cancer patients.” *Scientific Data* 6: <https://doi.org/10.1038/s41597-019-0131-5>

24. Wu, Thomas D., Shravan Madireddi, Patricia E. de Almeida, Romain Banchereau, Ying-Jiun J. Chen, Avantika S. Chitre, Eugene Y. Chiang, et al. 2020. “Peripheral T cell expansion predicts tumour infiltration and clinical response.” *Nature* 579: 274-278. <https://doi.org/10.1038/s41586-020-2056-8>

25. Zhang, Lei, Ziyi Li, Katarzyna M. Skrzypczynska, Qiao Fang, Wei Zhang, Sarah A. O’Brien, Yao He, et al. 2020. “Single-Cell Analyses Inform Mechanisms of Myeloid-Targeted Therapies in Colon Cancer.” *Cell* 181: 442-459.e429. <https://doi.org/10.1016/j.cell.2020.03.048>

26. Lee, Hae-Ock, Yourae Hong, Hakki Emre Etlioglu, Yong Beom Cho, Valentina Pomella, Ben Van den Bosch, Jasper Vanhecke, et al. 2020. “Lineage-dependent gene expression programs influence the immune landscape of colorectal cancer.” *Nature Genetics* 52: 594-603. <https://doi.org/10.1038/s41588-020-0636-z>

27. Luoma, Adrienne M., Shengbao Suo, Hannah L. Williams, Tatyana Sharova, Keri Sullivan, Michael Manos, Peter Bowling, et al. 2020. “Molecular Pathways of Colon Inflammation Induced by Cancer Immunotherapy.” *Cell* 182: 655-671.e622. <https://doi.org/10.1016/j.cell.2020.06.001>


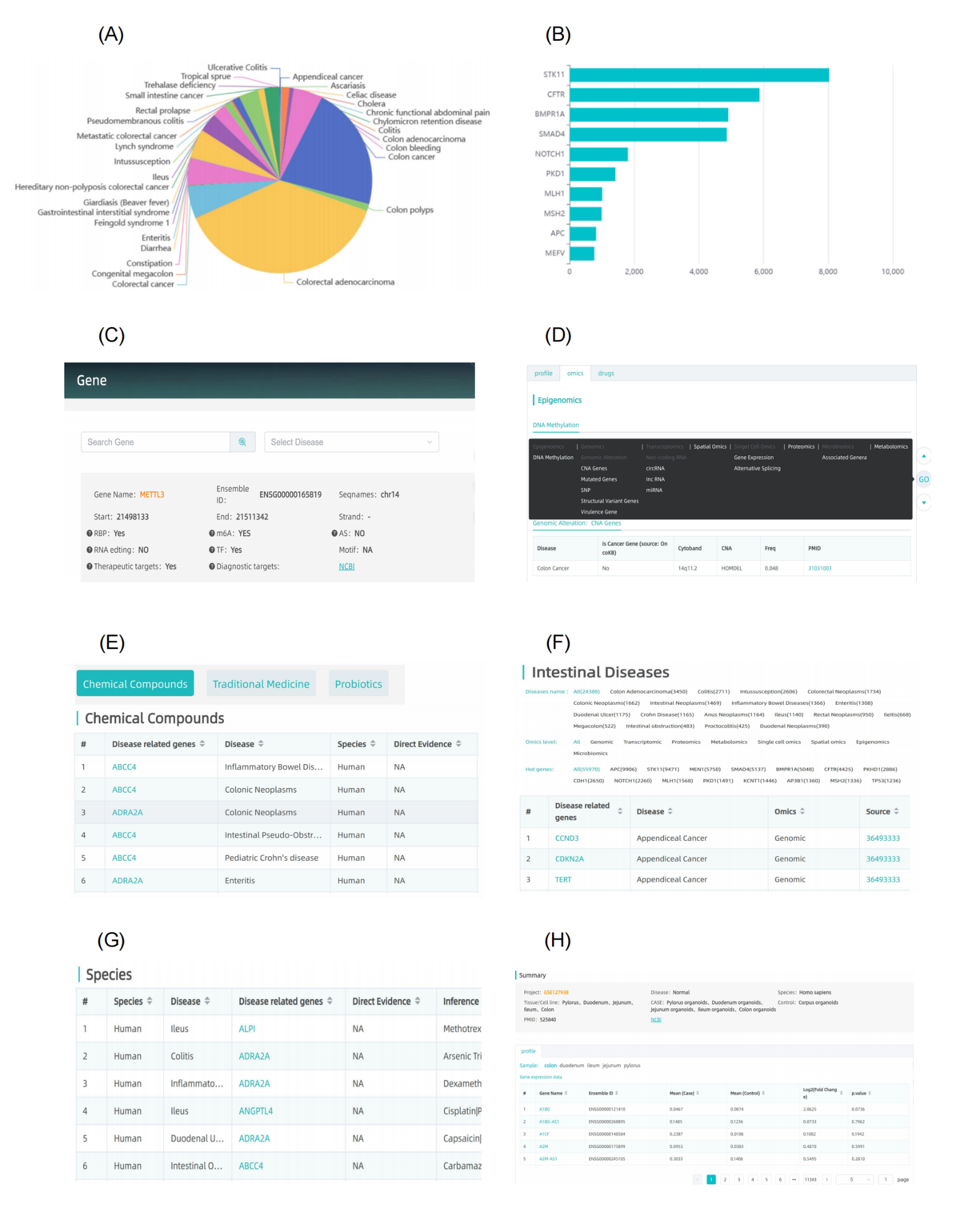


**Figure S1 Web display of content from other sections. (**A) The proportion of various intestinal diseases. (B) The top 10 genes associated with gene-disease. (C) The detailed gene content displayed after performing a search function. (D) Eight major omics information related to genes. (E) The therapy section contains chemical compounds, traditional medicine and probiotics. (F) Details page of the diseases section. (G) Details page of the species section. (H) The details page display of each dataset in genomics.
